# Risk group-4 virus emergent threats

**DOI:** 10.6026/973206300210721

**Published:** 2025-04-30

**Authors:** Paul Shapshak, Seetharaman Balaji, Charurut Somboonewit, John T. Sinnott, Francesco Chiappelli

**Affiliations:** 1 Department of Internal Medicine, Morsani College of Medicine, University of South Florida, Tampa, Florida 33606, USA; 2 Department of Biotechnology, Manipal Institute of Technology, Manipal Academy of Higher Education, Manipal, 576104, Karnataka, India; 3 UCLA Center for the Health Sciences, Los Angeles, California 90095, USA

**Keywords:** World Health Organization (WHO), NIH, NIAID, CDC, risk group 4 (rg-4) virus pathogens, filovirus (Ebola, Marburg and Sudan viruses), biosafety laboratory (BSL)-4, emergent virus, global warming, ecology, vector, reservoir, virulence, case fatality rate, Africa

## Abstract

Several Filovirus outbreaks occurred during 2021-2025 in Africa. The case fatality ratios (CFRs) were elevated as expected for these viruses. After outbreaks were termed terminated, nonetheless, due to virus pathogenicity and transmissibility, epidemics continued unabated. To mitigate future outbreaks and bolster pandemic preparedness, collaborative global strategies should continue and expand.

## Background:

Recent prior reports indicated the severe characteristics and consequences of Risk Group-4 (RG-4) virus outbreaks [1, 2]. The increasing incidence of RG-4 viruses poses significant public health challenges due to their lethality, limited treatment options and potential for efficient and swift human-to-human transmission. Continued climate change (global warming), vector spread, deforestation and urbanization are accelerating zoonotic spillovers, raising the likelihood of pandemics. Continued collaborative global strategies are crucial to bolster pandemic preparedness and mitigate future outbreaks. In brief, RG-4 viruses, which include the Filoviruses (*i.e.*, Filoviridae family), cause the most dangerous and lethal diseases in humans and require the maximum biosafety level 4 for biocontainment. Filoviruses consist of a family of single-stranded negative-sense RNA viruses whose representative includes Sudan, Ebola and Marburg viruses, among others [1, 2-3].

## Sudan virus disease outbreak in south Sudan (2021):

In December 2021, South Sudan reported an outbreak of Sudan virus disease. The outbreak involved three confirmed cases, with two deaths. It was declared over in February 2022 [4].

## Sudan virus disease outbreak in Uganda (2022):

On September 20, 2022, Uganda confirmed an outbreak of Ebola virus disease caused by the Sudan virus strain. As of January 11, 2023, there were 164 confirmed cases and 77 deaths, resulting in a CFR of 47%. The outbreak was declared over on January 11, 2023 [5].

## Ebola virus disease outbreak in Democratic Republic of the Congo (North Kivu Province) (2022):

On August 17, 2022, the Democratic Republic of the Congo (DRC) confirmed a new outbreak of Ebola virus disease in North Kivu province. There was one confirmed case, which was fatal. The outbreak was declared over on September 27, 2022. On August 21, 2022, the Democratic Republic of the Congo (DRC) confirmed the Ebola Virus Disease case in the North Kivu province. The case was linked to an earlier outbreak from 2018-2020, with genomic sequencing confirming it was caused by a persistent infection from a survivor rather than a new spillover event. As of September 27, 2022, the case was confirmed, resulting in death. The outbreak was declared over, after no new cases were reported for 42 days [6].

## Ebola virus disease outbreak in Democratic Republic of the Congo (Equateur Province) (2022):

On April 23, 2022, the DRC confirmed an outbreak of Ebola virus disease in Equateur Province. A total of five cases were reported, all of which were fatal. The outbreak was declared over on July 4, 2022 [7].

## Marburg virus disease outbreak in Equatorial Guinea (2023):

On February 13, 2023, Equatorial Guinea's Ministry of Health confirmed it's first-ever outbreak of Marburg virus disease. As of April 21, 2023, there were 17 laboratory-confirmed cases, including 12 deaths, resulting in a case fatality ratio (CFR) of 71%. The outbreak was declared over on May 15, 2023, after 42 days without new confirmed cases [8].

## Marburg virus disease outbreak in Tanzania (2023):

On March 21, 2023, Tanzania reported an outbreak of Marburg virus disease in Bukoba District, Kagera Region. A total of nine cases were reported, including eight deaths, resulting in an 89% case fatality ratio. The outbreak was declared over on June 2, 2023 [9].

## Marburg virus disease outbreak in Equatorial Guinea (2023):

The 2023 Marburg virus disease outbreak in Equatorial Guinea marked the country's first encounter with this severe hemorrhagic fever. The outbreak was officially declared on February 13, 2023, following reports of suspected viral hemorrhagic fever deaths between January 7 and February 7, 2023. By June 7, 2023, a total of 17 confirmed cases and 23 probable cases were reported across five districts in four provinces. Among the confirmed cases, 12 resulted in death and all probable cases were fatal, indicating a high case fatality rate. The outbreak was declared over on June 8, 2023, after 42 consecutive days without new confirmed cases [10].

## Marburg virus disease outbreak in Rwanda (2024):

On September 27, 2024, Rwanda's Ministry of Health confirmed the country's first-ever outbreak of Marburg virus disease (MVD). As of December 19, 2024, there were 66 confirmed cases and 15 deaths, resulting in a case fatality ratio (CFR) of 23%. The outbreak was declared over on December 20, 2024, after 42 days without new confirmed cases. A report from The Lancet, dated October 14, 2024, indicated 62 reported cases and 15 deaths, emphasizing the need for immediate public health interventions [11, 12].

## Marburg virus disease outbreak in Tanzania (2025):

On January 20, 2025, Tanzania's Ministry of Health confirmed an outbreak of Marburg virus disease in the Kagera Region. As of January 11, 2025, there were nine suspected cases, including eight deaths, resulting in a case fatality ratio of 89%. Samples from two patients were collected and tested by the National Public Health Laboratory, with results pending official confirmation. On March 13, 2025, the World Health Organization (WHO) reported that Tanzania declared the end of the Marburg virus outbreak after recording no new cases over 42 days since the death of the last confirmed case on January 28, 2025. A total of 10 cases were reported, including two confirmed and eight probable cases; all cases resulted in deaths [13].

## Marburg virus disease in Tanzania (2025):

An article from Gavi (The Vaccine Alliance), published three weeks ago, noted that all 10 Marburg patients in Tanzania had died, resulting in a 100% case fatality rate. No new cases had been reported in the weeks since [14].

## Key takeaway points:

## Recent upsurges in RG-4 viruses:

Several Filovirus outbreaks occurred in African countries, 2021-2025, with high case fatality rates, underpinning the up-surging threat of filoviruses.

## Challenges in outbreak control:

Rapid responses, detection, healthcare infrastructure and funding are crucial efforts for containment and needed to reduce the risk of amplified spread. Vaccines and anti-virals against Filoviridae are presently limited.

## High mortality and global health implications:

RG-4 viruses, including Sudan, Ebola and Marburg virus diseases, sustain alarming fatality rates (up to 100%). Consequently, critical advances in diagnostics, vaccines and antiviral therapies to reduce mortality are needed and ongoing.

## Epidemiological modeling for future preparedness:

Methods including Fourier Transform analysis, nonlinear dynamics and complex systems modeling can improve outbreak predictions; optimize containment strategies, Artificial Intelligence (AI) to enhance pandemic preparedness for future RG-4 virus outbreaks [15].

## Funding gaps and policy challenges:

While NIH, CDC, WHO and private foundations provide funding, resource allocation remains inadequate for RG-4 virus diseases in Africa, stressing the need for greater global investment in RG-4 virus research, response and preparedness.

## Conclusion and future directions:

Since the outbreaks described in this paper were all Filoviridae, it is to be noted that two publications by Kuhn and colleagues, provide comprehensive reviews and definitive Filoviridae history, classification, taxonomy, pathology and molecular biology. International teamwork in epidemiology research and the clinic is essential for halting the outbreaks and spread of RG-4 viruses. Analytic methods including Fourier Transform and complex systems modeling are needed. Vaccine development as well as molecular manipulations is crucial in such efforts. Funding for epidemiological and vaccine programs are crucial efforts to be sustained. Global socio-economic amplified measures are needed as well, to bolster efforts already underway. Collaborative global strategies are fundamental to mitigate future outbreaks and bolster pandemic preparedness [15, 16, 17, 18- 19].

Initial Fourier Transform analysis of the Filovirus outbreaks 2021 - 2025, revealed distinguishable spectral signatures for Ebola, Marburg and Sudan virus outbreaks in Africa (using previously published methods [20, 21]. The temporal incidence signals for each virus showed dominant low-frequency components corresponding to annual or biennial recurrence, with Marburg exhibiting the sharpest periodic peak near a 1-year cycle. Smoothing and spectrogram overlays suggested potential seasonal clustering, particularly for Marburg and Sudan virus outbreaks. The Fourier Transform analysis also indicated minimal cross-virus temporal overlap within individual countries, hinting at competitive or interference dynamics rather than synergistic spread. These findings, though preliminary, support the utility of Fourier Transform in identifying hidden temporal structures and potential seasonality in outbreak dynamics. Future studies will use broader time-frequency methods and extend to pre- and post-SARS-2 pandemic filovirus outbreak data in Africa.
